# GenX uptake by wheat and rice in flooded and non-flooded soils: a greenhouse experiment

**DOI:** 10.1007/s11356-023-31160-w

**Published:** 2023-12-04

**Authors:** Amnah Al Zbedy, Viktoria Müller, Andrew Kindness, Rainer Ebel, Gareth J. Norton, Joerg Feldmann

**Affiliations:** 1 School of Biological science, University of Aberdeen, Machar Drive, Aberdeen, AB24 3FX UK; 2 School of Natural and Computing Sciences, University of Aberdeen, Meston Walk, Aberdeen, AB24 3UE UK; 3 Department of Chemistry, Al-Qunfudhah University College, Umm Al-Qura University, Makkah, Saudi Arabia; 4 TESLA-Analytical Chemistry, University of Graz, Universitätsplatz 1, 8010 Graz, Austria; 5 The James Hutton Institute, Craigiebuckler, Aberdeen, AB15 8QH UK; 6 School of Chemistry and Physics, University of KwaZulu-Natal, Private Bag X54001, Durban, 4000 South Africa

**Keywords:** PFAS, Plant uptake, Rice, Agriculture practise, Wheat, EOF

## Abstract

GenX (hexafluoropropylene oxide dimer acid) belongs to the group of per- and poly-fluoroalkyl substance (PFAS) compounds introduced to replace perfluorooctanoic acid (PFOA), which has been phased out in industrial and consumer product formulations. While GenX has been investigated in lab animals, there is limited information available regarding its uptake and translocation in wheat and rice. This study reports on a greenhouse experiment in which wheat and rice grown under flooded and non-flooded conditions were exposed to two GenX concentrations in the soil (0.4 mg kg^−1^ and 2 mg kg^−1^). GenX was analysed in the soil, porewater and shoots using targeted liquid chromatography-tandem mass spectroscopy (LC-MS/MS) analysis. Extractable organic fluorine (EOF) was determined using high-resolution continuum source graphite furnace molecular absorption spectrometry (HR-GFMAS) instrument. Results showed that different species took up different amounts of GenX. The GenX concentration in rice shoots was found to be 2.34 (± 0.45) and 4.11 (± 0.87) μg g^−1^ under flooded and non-flooded conditions, respectively, at a low exposure level. At high exposure, the GenX concentrations in flooded and non-flooded rice shoots increased threefold to 10.4 (± 0.41) and 13.4 (± 0.72) μg g^−1^, respectively. Wheat shoots showed similar concentrations and increases between low- and high-level exposure. The translocation factor was significantly higher (*P* = 0.013) in non-flooded rice compared to flooded rice. The GenX bioaccumulation behaviours under the same culture conditions (e.g. temperature, humidity, light, same GenX concentration in the soil) were significantly different in non-flooded and flooded rice (*P* < 0.001). Non-flooded rice plants displayed a higher level of GenX bioaccumulation than flooded ones. Following exposure to GenX, flooded rice plants showed a reduction in biomass (25%) compared to the control plants (*P* < 0.014). Our findings indicate that GenX is a bioaccumulative compound, the presence of which likely inhibits the growth of plants.

**Supplementary Information:**

The online version contains supplementary material available at 10.1007/s11356-023-31160-w.

## Introduction

Hexafluoropropylene oxide dimer acid (HFPO-DA), a perfluoroalkyl ether carboxylic acid also known as GenX, has been used to aid polymerisation in the production of high-performance fluoropolymers (Bokkers et al. 2018). It was introduced as an alternative for perfluorooctanoic acid (PFOA), which was used from 1970 to 2012 (Rivm and Poll). Organisations such as the Stockholm Convention and the European Chemical Agency began investigating PFOA as a hazardous contaminant due to its persistence, bioaccumulation and toxicity (Brandsma et al. 2019), leading to its restriction in 2017 by the European Union (Brendel et al. 2018). This restriction gave rise to so-called replacement chemistries, including GenX, which was introduced for its lower bioaccumulation potential compared to PFOA, despite the limited toxicokinetic data available for GenX (Heydebreck et al. 2015; Zhang et al. 2021). The United States Environmental Protection Agency (US-EPA) recently published toxicity assessments for PFOA and perfluorooctanesulfonic acid PFOS in 2016, showing that the chronic oral reference doses (RfDs) for PFOS and PFOA were higher than those for GenX. Based on available animal toxicity data, GenX appears to be more hazardous than PFOA and PFOS PFOS (Wang et al. 2015; Gomis et al. 2018; US Environmental Protection Agency 2021). GenX has effects in mice and zebrafish (Satbhai et al. 2022), such as the induction of benign tumours, gene changes in the liver, thyroid hormone level disturbances and hepatocellular damage (Cannon et al. 2020).

Increasing amounts of GenX have been detected in drinking water and soil (Gebbink and van Leeuwen 2020). In 2015, GenX contamination was discovered near the Cape Fear River in North Carolina, downstream of a chemical manufacturing company (Cahoon 2019). GenX was also detected in plant leaves within 3 km of a fluoropolymer-manufacturing Teflon plant in the Netherlands (Brandsma et al. 2019). It is unclear how GenX and PFASs are absorbed by grass and foliage. The GenX concentrations observed in the grass and leaves around the plant may have resulted from air deposition, absorption from contaminated soil or both (Brandsma et al. 2019). Perfluoroether chains are similarly resistant to biotic and abiotic degradation as PFOA (Wang et al. 2015).

Although most studies have focused on the effects of GenX on animals, knowledge regarding GenX bioaccumulation and its adverse effects on plants is limited. Although studies on the uptake and accumulation of GenX by plants are limited, it is an increasing area of interest and investigation. Recently, the accumulation and toxicity of GenX and perfluorooctanoic acid (PFOA) have been studied and compared using *Arabidopsis thaliana* (L.) Heynh and *Nicotiana benthamiana* Domin as model plants in a hydroponic system. These findings indicated that at concentrations between 20 and 200 mg L^−1^, GenX inhibited plant growth and lowered chlorophyll content and enzyme activity (Chen et al. 2020). Zhang et al. (2021) found that *Carex comosa* Stokes absorbs nearly 8% of the GenX in soil after 80 days of exposure. Moreover, Zhi et al. (2022) studied the bioaccumulation of PFAS in spontaneous urban plants and reported that GenX had a lower bioaccumulation factor (0.66–2.5) than PFOA (3.5–10.5) in plant roots.

PFAS are typically analysed using targeted LC-MS/MS; however, only those that ionise easily by electrospray ionisation can be analysed. Extractable organic fluorine (EOF) or total fluorine analysis are broader PFAS analyses methods. Different instruments can be used for EOF and total fluorine analysis, such as combustion ion chromatography (CIC) and high-resolution graphite furnace molecular absorption spectrometry (HR-GFMAS). Although results obtained with the two different instruments are comparable (Gehrenkemper et al. 2021), HR-GFMAS analysis has been found to be more sensitive and less time consuming (Gehrenkemper et al. 2021). Mass balance analysis, meanwhile, combines the target and EOF analyses to identify the fraction of PFAS that can and cannot be determined using the target method (Aro et al. 2021).

Rice is one of the most representative foods among primary nutritional foods (Liu et al. 2018) and is consumed by more than 50% of people around the world (da Silva et al. 2018). Wheat is also a significant source of carbohydrates, fibre and vitamins worldwide (Shewry and Hey 2015). Although PFAS intake from rice and wheat consumption is a health concern, considering the uptake of legacy and replacement PFAS, only a few studies (Stahl et al. 2009; Lan et al. 2018; Kim et al. 2019) on the uptake and bioaccumulation of these chemicals in rice and wheat are available.

The present study used a mass balance approach to study the accumulation of GenX in plants grown in contaminated soil and to determine the contribution of GenX to EOF in wheat and rice. To the best of our knowledge, this study is the first to investigate GenX uptake by wheat and rice from soil systems and to measure the total EOF in wheat and rice using HR-GFMAS in addition to targeted LC-MS/MS.

## Material and methods

### Chemical reagents and laboratory materials

The chemical 2,3,3,3-tetrafluoro-2-(heptafluoropropoxy) propanoic acid GenX (97% purity) was obtained from SynQuest Laboratories. A PFAS standard solution (MPFAC-MXC) containing perfluorinated carboxylic acid (PFCA) and perfluoroalkanesulfonic acids PFSA was purchased from Wellington Laboratories. Ultrapure water with a resistivity of 18.2 MΩ cm was obtained from Smart2 Pure, Thermo Fisher Scientific (Loughborough, UK). Ammonium hydroxide (Merck) and HPLC-grade methanol (Honeywell Riedel-de Haen, Germany) were used for sample extraction, and Envi carb (Merck) was used for cleaning purposes. Acetonitrile (Honeywell Riedel-de Haen, Germany) was used to prepare the mobile phase for HPLC. For the HR-GFMAS analysis, BOC (Dublin, Ireland) provided 99.998% purity argon gas, and a W (Merck) standard solution was used for the graphite tube coating as a permanent modifier. Ca(NO_3_)_2_x4H_2_O (VWR chemicals, Leicestershire, UK) was used as a forming reagent at a concentration of 1% Ca (w/v). PFOA, which was used as a calibration solution, was obtained from Sigma-Aldrich (St Louis, MO, USA).

### Soil properties and treatment

Soil was collected from a field in Insch Aberdeenshire. The soil samples were collected from the top 10 cm of the field. The soil was air-dried for 2 weeks and sifted through a 2-mm sieve. Soil pH (7.29) was measured in deionised water with a soil-to-water ratio of 1:2.5 after shaking for 1 h (Table S1). The soil was then spiked with a stock solution of GenX to achieve two nominal concentrations of 0.4 mg kg^−1^ (low level) and 2.0 mg kg^−1^ (high level). The control soil was also spiked with methanol. Once the soils had been spiked, they were placed in a fume hood for solvent evaporation at room temperature. Once the solvent evaporated, the soil was incubated in darkness for 14 days at room temperature. Concentrations of GenX in the soil were determined after incubation but before adding plants to the soil (0 day) and at the end of the plant growth experiment (30 days).

### Plant exposure in soil and sampling

Plastic pots (0.9 L) were filled with 650 g of soil. Four groups of tests were conducted:

1. soil alone, spiked with water (control 1);
2. spiked soil alone, spiked with methanol (control 2);
3. and (4) spiked soil with low and high GenX concentrations, respectively.

Each group contained four replicates for each plant species and water treatment.

For the wheat and non-flooded rice experiments, a piece of filter paper was placed at the bottom of each pot to restrict soil loss. The pots were lined with plastic bags for the flooded rice experiment to allow the soil to be flooded without loss. Wheat (Tybalt) and rice (BJ 1) seeds were germinated for 3 days on a petri dish, with the wheat at room temperature and the rice at 25 °C. Ten seeds were transplanted into each pot. The pots were irrigated daily to maintain a moisture content at 80% water-holding capacity for the wheat and non-flooded rice whereas the flooded rice was saturated until standing water was maintained. The pots were randomly positioned daily to account for spatial variations in light and temperature within the greenhouse. After 20 days, 2 mL of 10× Yoshida’s nutrient solution (Yoshida 1976) was added. Rice and wheat were grown in separate greenhouses for 4 weeks at the University of Aberdeen in Aberdeen, Scotland (coordinates: 57.165°N 2.100°W). For the rice experiment, the day temperature was 28 °C and the night temperature 25 °C. Supplemental lights were turned on between 7 am and 7 pm if the natural light was less than 24,000 lux. The wheat experiment was kept at 15 °C.

After 30 days, the shoots of wheat and rice were harvested, freeze-dried and homogenised, then stored in polypropylene (PP) vials at − 20 °C prior to analysis.

Following the removal of the plants from each pot, all test soils were taken out and air- and freeze-dried for 48 h at room temperature. Before chemical analysis, the dried soil samples were stored in polypropylene (PP) tubes at − 20 °C.

### Measurements of plant height and porewater collection

Rhizon samplers attached to a 10-mL syringe were buried at a 45° angle in the soil to allow sampling of porewater. Samplers of the porewater was taken on first week transplanting (day 0) and at the end of experiment at day 30 for rice. Plant growth was determined by measuring plant height, dry shoot biomass and number of tillers. The measurements were performed on day 30. Shoots were cut at 3 cm above the soil level to avoid soil contamination and its dry weight biomass were determined using an analytical balance.

### Analysis and extraction of PFAS

#### Extraction procedure

The GenX in the plant was extracted using a previously described method (Blaine et al. 2013; Chen et al. 2020). As an extraction solvent, a mixture of 50:50 (v/v) dichloromethane (DCM) and methanol (MeOH) with 1% ammonium hydroxide (v/v) was prepared. For the extraction, 1 mg of dried plant sample was placed into a 15-mL polypropylene tube, and 3 mL of the extraction solvent was added. The tube was then vortexed for 30 s, sonicated for 15 min at 30 °C, then shaken on an orbital shaker for 1 h. The supernatant was collected after centrifugation at 1500 rpm for 10 min. This extraction process was repeated twice. The extracts were then pooled and dried under a gentle nitrogen stream. The dried extract was reconstituted with 1 mL of MeOH, then mixed with 50 mg of ENVI-Carb for 20 s for clean-up. This was followed by centrifugation at 13,000 rpm for 10 min. Then, 450 μL of the extract was transferred into HPLC vials. Finally, 50 μL of the 50 μL L^−1^ isotopically mass-labelled standard was introduced to enable target analysis using LC-MS/MS.

Soil samples were extracted by transferring a 0.5 g aliquot into a 50-mL polypropylene vial, to which a solution containing an isotopically labelled surrogate standard and 3 mL of 0.1% ammonium hydroxide (NH_4_OH) in methanol was added. This was then sonicated for 15 min at 30 °C, as per the established protocols (Higgins et al. 2005; Houtz et al. 2013; Zhang et al. 2021). The solution was then shaken for 1 h on an orbital shaker. The supernatant was collected after centrifugation at 1500 rpm for 10 min. This extraction process was repeated twice. All extracts were combined, dried under a gentle nitrogen stream, reconstituted in 1 mL methanol and cleaned with 50 mg ENVI-Carb. Then, 450 μL was taken into HPLC vials, and 50 μL of the 50 μL L^−1^ isotopically mass-labelled standard was added for target analysis using LC-MS/MS.

The porewater samples were analysed directly via LC-MS/MS. Prior to injection, each vial was centrifuged for 20 min at 2700 rpm. An aliquot (225 μL) of the supernatant was then removed and transferred to a PP microcentrifuge tube containing 250 μL of methanol. Then, 50 μL of the 50 μL L^−1^ isotopically mass-labelled standard was added for target analysis using LC-MS/MS.

### Instrumentation

#### Target PFAS

Agilent 1200 infinity HPLC (Agilent Technologies, Germany), coupled with Agilent 6465 Triple Quadrupole MS/MS (Agilent Technologies, Germany), operated in negative electrospray ionisation (ESI) mode. PFAS separation was performed using 5 mM ammonium acetate (CH_3_COONH_4_) in reagent water, and 100% LCMS-grade acetonitrile was used as the mobile phase of the analysis of PFAS ionic compounds. Ten μL of the extract was automatically injected onto a BrownLee SPP C18 column (2.7 μm, 3 × 100 mm, PerkinElmer, UK) at 30 °C. Chromatograms were recorded using multiple reaction monitoring (MRM) mode. A list of the analytes, transitions and optimised MRM conditions and LC method used can be found in the Supplementary Materials (Table S2-S4).

#### EOF

The EOF were analysed according to the method described by Akhdhar et al. (2019). All measurements were performed using an Analytik Jena ContrAA 700 High Resolution Continuum Source Graphite Furnace Atomic Absorption Spectrometer (HR GFAAS) with a transversely heated graphite tube atomiser. A 300-W xenon short-arc lamp was used as the instrument’s continuous radiation source for wavelengths between 185 and 900 nm. A charge-coupled device (CCD) array detector with 588 pixels, 200 and a high-resolution double echelle monochromator were used for analytical purposes. The measurements were conducted using coated graphite tubes with an integrated PIN platform (Analytical Jena part No. 407-A81.025). All fluorine measurements were performed three times at a wavelength of 606.417 nm to monitor the absorption of the formed CaF. The graphite furnace platform was coated with tungsten (W), while calcium (Ca) was utilised as the forming reagent. Table S5 details the temperature programme used.

#### Fluorine mass balance analysis

Fluorine mass balance analysis was conducted by converting the concentration of PFAS analyte in the samples measured by LCMS/MS into the corresponding fluorine concentrations equivalents. This was then compared to the fluorine concentrations obtained from the EOF analysis using HR-GFMAS.

### QC and statistical analysis

#### Targeted LC-MS/MS analysis

A 4- to 5-point calibration curve in 50% (v/v) methanol, ranging from 0.05–0.1 to 5–20 g kg^−1^, was prepared prior to the run. To evaluate instrumental drift, three calibration blanks were processed with extra higher and lower quality control (QC) standards at concentrations of 0.5 μg kg^−1^ and 5 μg kg^−1^, respectively. The standard error of the y intercept of the linear regression line was used as the basis for calculating the limits of detection and quantification (LOD and LOQ). LOD and LOQ were calculated at 3 and 10 times the *y* intercept error and found to be 2.31 μg kg^−1^ and 7.72 μg kg^−1^, respectively.

#### Total EOF

A calibration curve was prepared using PFOA in 50% (v/v) methanol ranging from 0 to 2000 μg F L^−1^. Ten blank measurements were conducted using deionised water, then the blank standard deviation (SD) was calculated, and the result was divided by the slope of the calibration curve, which was taken on the same day of blank measurement, multiplied by 3 to obtain the value of the instrumental LOD. The LOD calculated to be 0.49 μg F L^−1^.

### Statistical analysis

Data were expressed as the mean ± standard deviation (SD) on a dry weight (dw) basis. The statistical analysis included one- and two-way analysis of variance (ANOVA) approaches. All analyses were performed using Minitab 20 (Minitab LLC, USA) and Microsoft Excel (Microsoft, USA) software. The least significant differences (*P* < 0.05) were determined and used to compare the statistical significance between treatments.

## Results

### Effects of GenX on the growth of rice and wheat

**Fig. 1:**
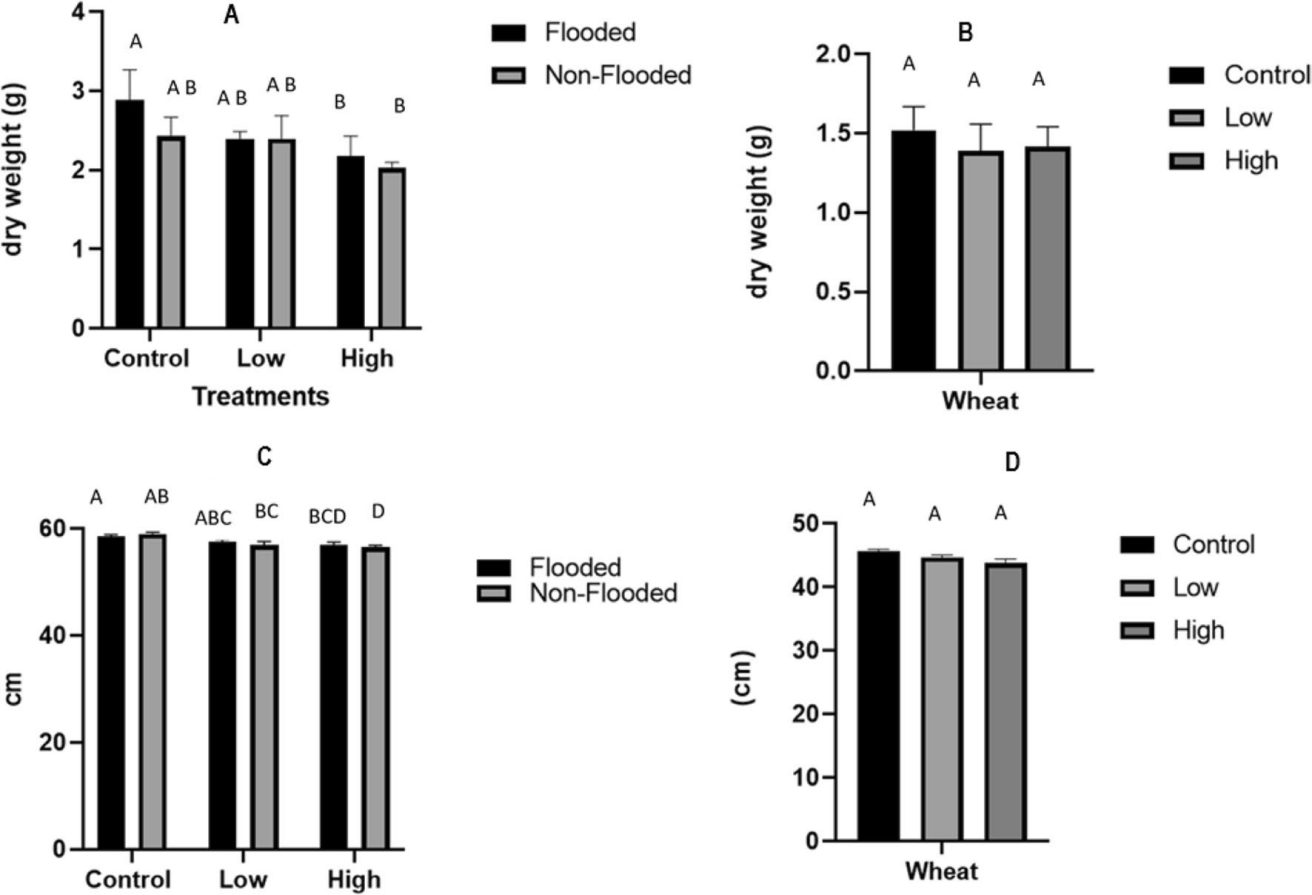
Treatment of rice (**A** and **C**) and wheat (**B** and **D**) biomass (g) and shoot length (cm). The error bars represent 1 standard deviation. Different letters represent significant differences among the treatments; means that do not share a letter are significantly different

Growth characteristics, including shoot biomass and length of wheat and rice crops, under GenX application are presented in (Fig. 1). The shoot length of wheat showed non-significant (*P* > 0.05) results for both GenX treatments compared to the control with *P* = 0.762 (Table 1). The rice shoots’ dry biomass, however, exhibited a significant reduction of 25% (*P* = 0.02) at 2 mg kg^−1^ GenX concentrations compared to the control under flooded soil conditions (Fig. 1). Further, reductions of 9% and 7% were observed for the rice shoots’ dry biomass at 0.4 and 2 mg kg^−1^ GenX concentrations, respectively, compared to the non-flooded control. The flooded and non-flooded soil conditions exhibited non-significant (*P* = 0.096) effects on the rice shoots’ dry biomass at both GenX concentrations (Table 2). Rice plant height, however, was significantly affected (*P* = 0.048) under varied soil conditions. Rice shoot length showed a significant reduction with GenX and with varied soil environments (flooded (*P* 0.048) and non-flooded (*P* = 0.001), indicating that the percentage of reduction increases with higher GenX concentrations.

**Table 1:** Statistical analysis of GenX concentrations in wheat under varying treatment conditions

Parameters	GenX treatment
Biomass	*P* = 0.451
Plant height	*P* = 0.762
Number of tillers	*P* = 0.178
Shoot GenX conc.	*P* = 0.001

**Table 2:** Statistical analysis of GenX concentrations in rice under varying treatment conditions

Parameters	GenX treatment	Growth conditions	GenX growth conditions interaction
Biomass	*P* = 0.001	*P* = 0.096	*P* = 0.436
Plant height	*P* = 0.001	*P* = 0.048	*P* = 0.091
Number of tillers	*P* = 0.301	*P* =1	*P* = 0.017
Shoot GenX conc.	*P* = 0.001	*P* = 0.003	*P* = 0.349

The number of rice tillers exhibited a non-significant effect with GenX exposure (*P* = 0.301) and under varied soil conditions (*P* = 1). The interaction between GenX treatments and soil conditions is only significant for the number of tillers, with a *P* value (*P* = 0.017).

Overall, the rice crops grown under non-flooded soil exhibited a lower tolerance to GenX treatments compared to the wheat crops.

### Uptake of GenX into shoots

**Fig. 2:**
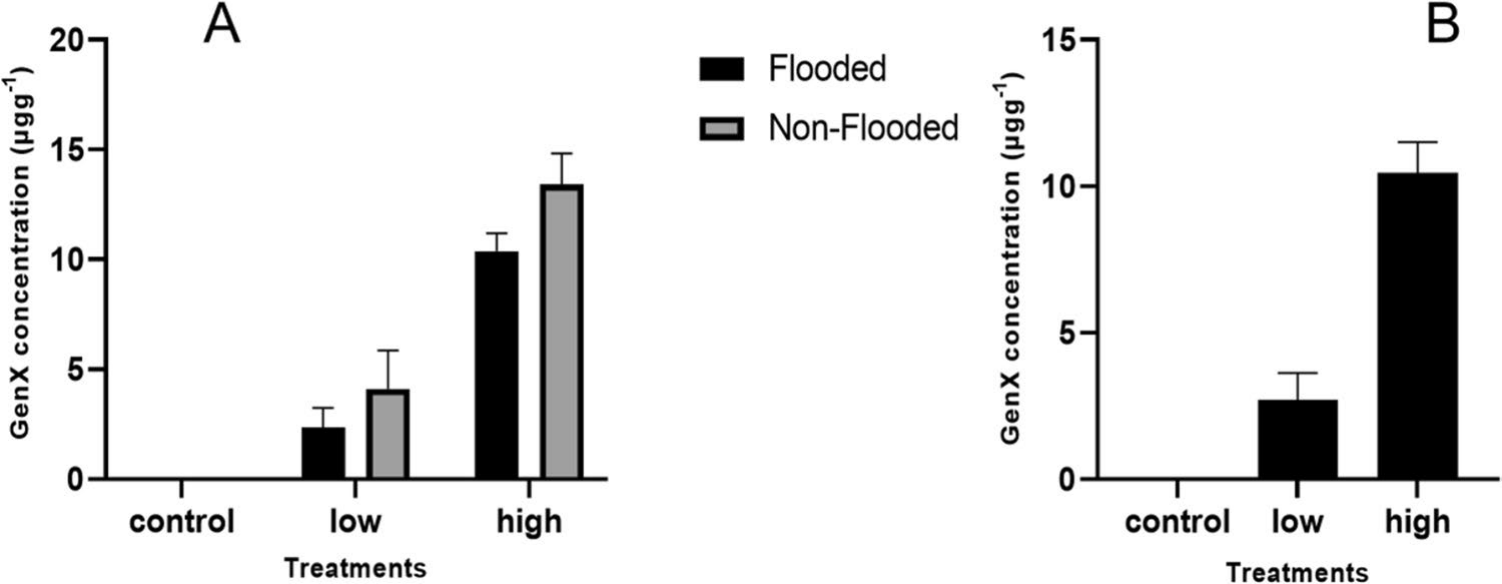
Concentration of GenX in rice (**A**) and wheat (**B**) shoots exposed to GenX at 0.4 mg kg^−1^ (low) and 2 mg kg^−1^ (high) after 30 days

The concentration of GenX significantly varied (*P* ˂ 0.05) with the GenX treatments and under different soil conditions (Fig. 2). The uptake of GenX increased as the level of GenX treatment increased in both tested crops. After 30 days of GenX exposure, the wheat shoot’s uptake was 2.71 (± 0.92) and 10.5 (± 1.05) μg g^−1^ at 0.4 and 2.0 mg kg^−1^ of GenX application, respectively. This concentration of GenX in the plant shoot was significantly higher than that of the control group. The control soil had a GenX concentration below LOQ.

The rice plants showed significantly varied GenX concentration values in their shoots according to the different soil conditions. The maximum uptake of GenX was observed in non-flooded soil conditions over flooded (*P* < 0.003). At 0.4 mg kg^−1^ of GenX, the rice shoots exhibited 2.34 (± 0.45) and 4.11 (± 0.87) μg g^−1^ of GenX uptake in flooded and non-flooded soil environments, respectively. When exposed to 2.0 mg kg^−1^ of GenX, the rice shoots showed a GenX uptake level of 10.4 (± 0.41) μg g^−1^ under flooded conditions and 13.4 (± 0.72) μg g^−1^ under non-flooded conditions. The uptake of GenX by the rice shoots showed a non-significant effect under the interactivity of the GenX treatment and soil condition (*P* = 0.34). Overall, the rice shoots in non-flooded soil conditions exhibited more GenX uptake when exposed to higher concentrations of GenX than did the wheat shoots.

**Fig. 3:**
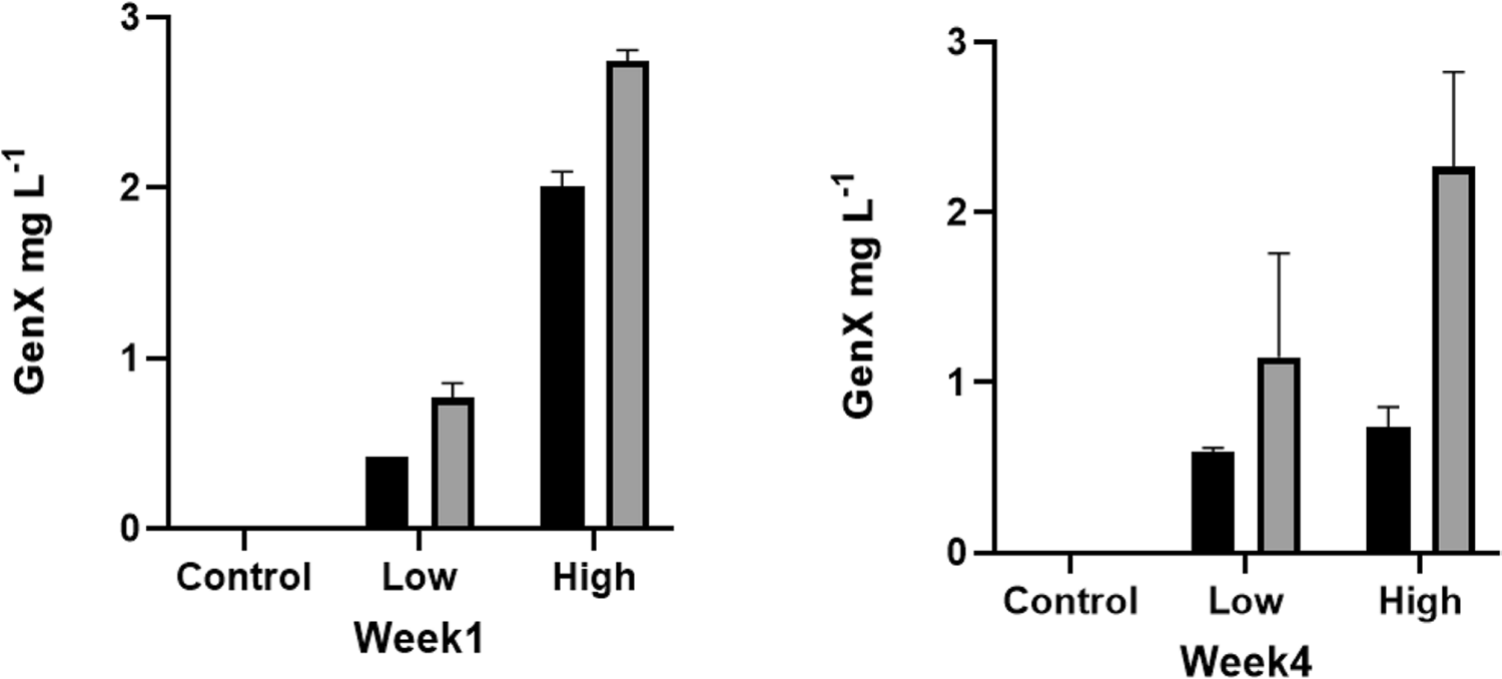
Concentration of GenX in porewater under flooding (black bar) and non-flooding (grey bar) condition exposed to GenX at 0.4 mg kg^−1^ (low) and 2 mg kg^−1^ (high) at the start and the end of rice growth experiment

The mass of GenX taken up by plant tissues (Fig. 1S, Fig S2) was calculated by multiplying the plant dry biomass by the GenX concentration. Overall, GenX mass in rice and wheat shoots had a distribution pattern similar to that of GenX in plant tissues. Across all experiments, non-flooded rice had the highest GenX mass compared to wheat. Furthermore, the comparison between flooded and non-flooded rice revealed a noticeable increase in GenX concentration in non-flooded rice particularly in pore water analysis as shown in (Fig. 3).

**Fig. 4:**
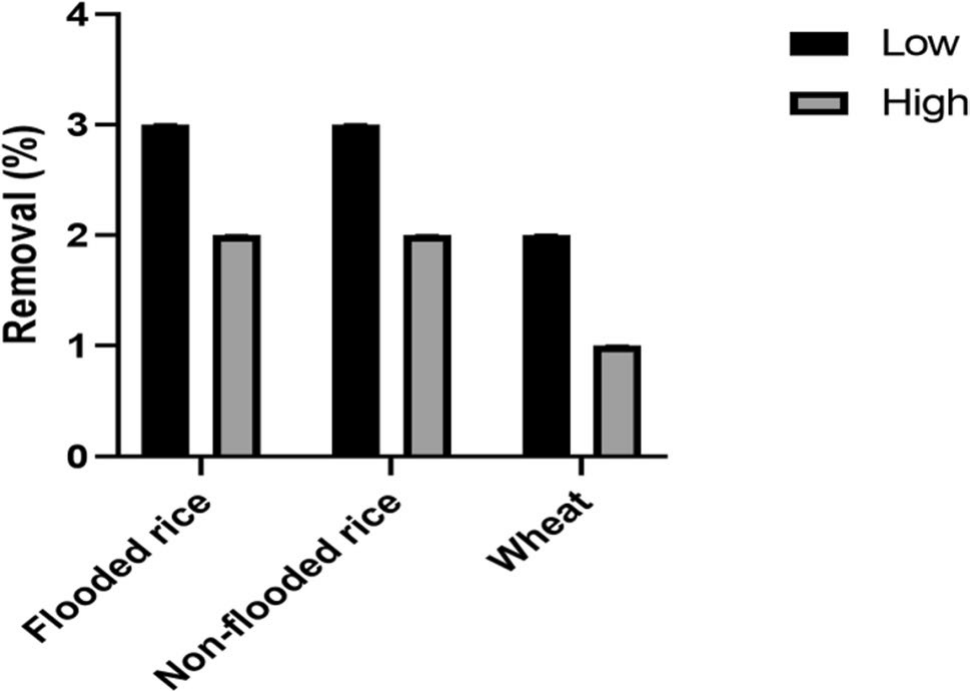
Removal of GenX in soil by plant (translocation of GenX from soil to plants) after 30 days. Error bars represent standard deviations (*n* = 4)

The removal efficiency (%) of the rice and wheat was significantly (*P* ˂ 0.05) affected by the GenX application level and soil conditions. This was calculated by dividing the mass of the GenX in the plant tissues by the mass of spiked GenX (Fig. 4). Generally, a higher removal percentage was observed at 0.4 mg kg^−1^ of GenX exposure with respect to the 2.0 mg kg^−1^ treatment. The wheat plants exhibited a removal efficiency of 2% and 1% with low and high concentrations of GenX, respectively, while the rice crops exhibited a removal efficiency of 3% and 2% at lower and higher GenX levels, respectively, in both soil environments.

### Distribution coefficient

The distribution coefficient, *K*_*d*_, describes the distribution of a chemical between two media or two phases. Prior studies have used this approach to evaluate the accumulation and mobility of PFAS in solid-water systems (Milinovic et al. 2015). Further, its value has been used to provide information on the distribution and final course of PFAS. The *K*_*d*_ value for the present study was determined as the ratio of the concentration of GenX measured in the soil phase and the concentration of GenX measured in the aqueous phase, as detailed below (Nguyen et al. 2020):

$$Kd\left(\frac{\textrm{L}}{\textrm{kg}}\right)=\frac{\ \textrm{Measured}\ \textrm{concentration}\ \textrm{in}\ \textrm{the}\ \textrm{soil}\ \textrm{phase}\ \left(\frac{\textrm{mg}}{\textrm{kg}}\right)}{\textrm{Measured}\ \textrm{in}\ \textrm{the}\ \textrm{aqueous}\ \textrm{phase}\ \left(\frac{\textrm{mg}}{\textrm{L}}\right)}$$

GenX exhibits low *K*_*d*_, which indicates that GenX is very mobile (Table 3). *K*_*d*_ levels with low and high concentrations of GenX were higher in the flooded rice than in the non-flooded rice (*P* = 0.028). Hence, GenX is significantly more mobile in the non-flooded environment.

**Table 3:** *K*_*d*_ values in rice under two different treatments. Errors are represented by 1 standard deviation for four replicates

Treatment	Flooded rice		Non-flooded rice	
	Week 1	Week 4	Week 1	Week 4
Low	0.54 (± 0.02)	0.04 (± 0.03)	0.35 (± 0.05)	0. 07 (± 0.02)
High	0.49 (± 0.10)	1.08 (± 0.34)	0.43 (± 0.02)	0.68 (± 0.23)

### TF

The translocation factor was used to describe the ability of the rice to translocate GenX to the plant shoots. The translocation factor (TF) values were calculated by the ratio of the concentration of GenX measured in the shoot and the concentration of GenX measured in porewater, as detailed below:

$$\textrm{TF}=\frac{\textrm{Measured}\ \textrm{concentration}\ \textrm{in}\ \textrm{the}\ \textrm{shoot}}{\textrm{Measured}\ \textrm{concentration}\ \textrm{in}\ \textrm{the}\ \textrm{porewater}}$$

The values for the translocation factor significantly varied under application of GenX (*P* = 0.001) and soil conditions (flooded and non-flooded) (*P* = 0.02) (see Table 4). Maximum TF values were observed at 2.0 mg kg^−1^ of GenX treatment, not at the 0.4 mg kg^−1^ treatment. TF values of 3.08 (± 0.12) and 3.80 (± 0.35) were observed under the flooded and non-flooded conditions, respectively, at low exposure. At high exposure, the TF values were observed at 5.00 (± 0.07) and 5.33 (± 0.14) in the flooded and non-flooded environments, respectively. These higher TF values in the non-flooded environment indicate that rice plants can translocate more effectively in non-flooded conditions than in flooded conditions.

**Table 4:** Translocation factor (TF) values in rice under two different growing conditions. Error represents 1 SD (*n* = 4)

Treatment	Flooded rice	Non-flooded rice
GenX at 0.4 mg kg^−1^	3.08 (± 0.12)	3.80 (± 0.35)
GenX at 2 mg kg^−1^	5.00 (± 0.07)	5.33 (± 0.14)

### Total EOF measurement and mass balance analysis

**Fig. 5:**
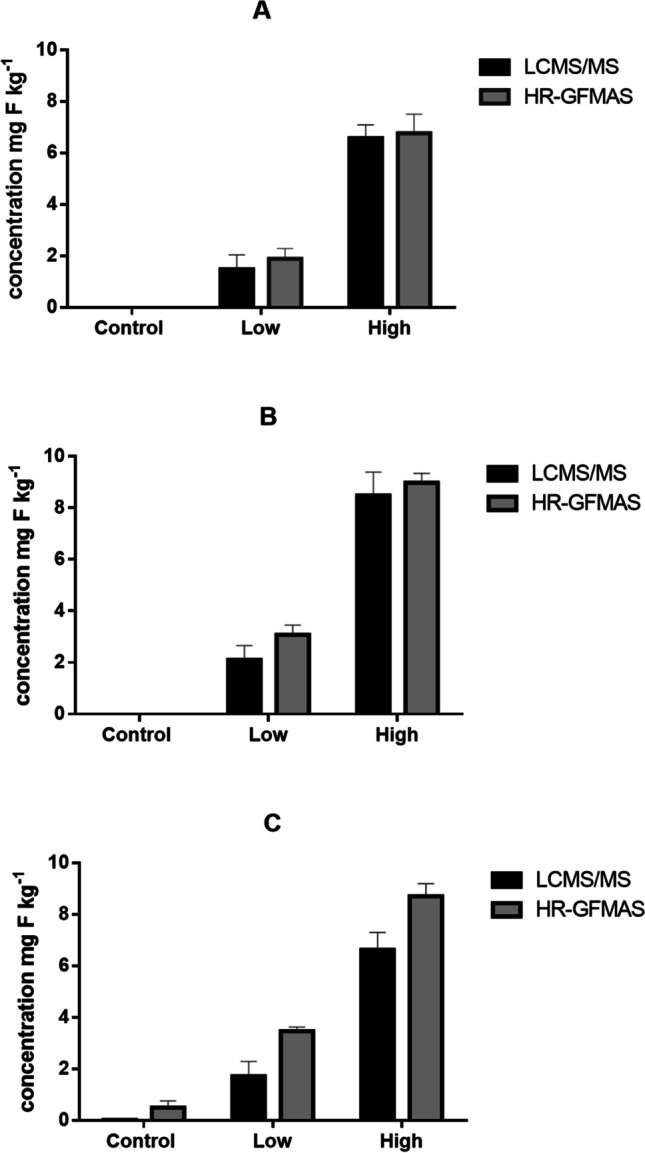
Fluorine mass balance analyses of rice: **A** flooded, **B** non-flooded and **C** wheat shoots. The black bars represent the contribution of GenX mg F kg^−1^ using LCMS/MS, while grey bars represent the total extractable organic fluorine using HR-GFMAS. Error bars represent by standard deviations (*n* = 4)

**Fig. 6:**
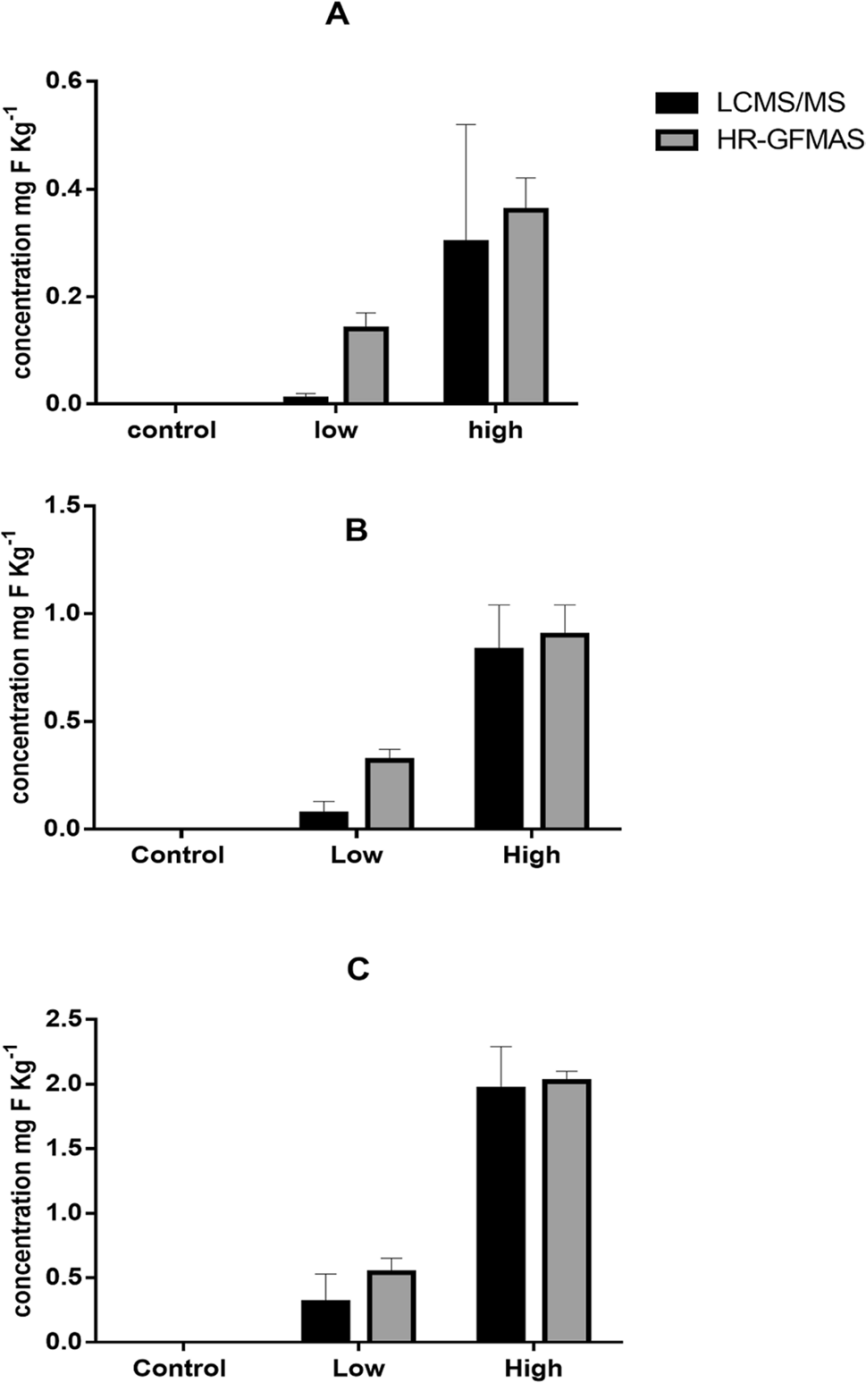
Fluorine mass balance analyses of soil: **A** flooded, **B** non-flooded and **C** wheat. The black bars represent the contribution of GenX mg F kg^−1^ using LCMS/MS, while grey bars represent the total extractable organic fluorine using HR-GFMAS. Error bars represent standard deviations (*n* = 4)

**Fig. 7:**
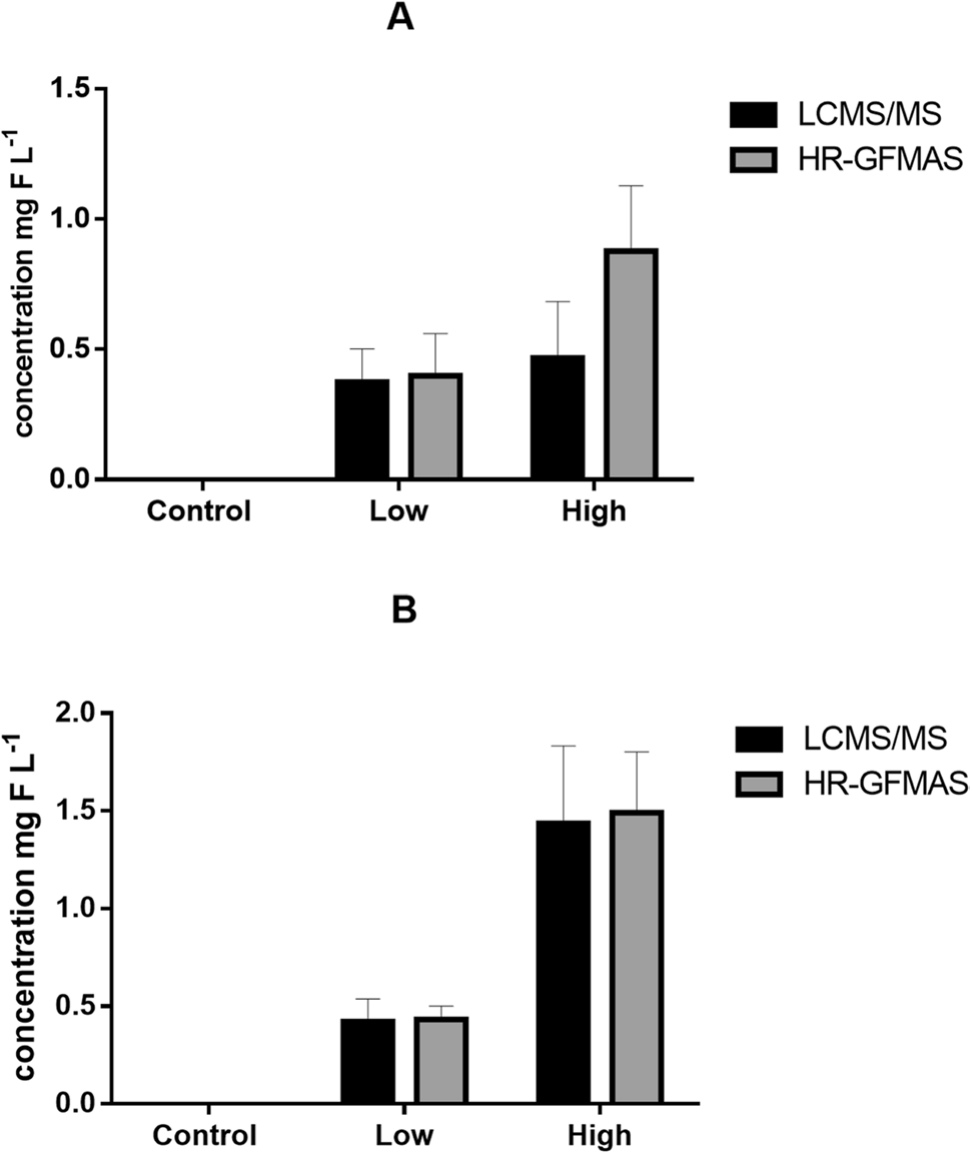
Fluorine mass balance analyses of porewater: **A** flooded, **B** non-flooded. The black bars represent the contribution of GenX mg F L^−1^ using LCMS/MS, while grey bars represent the total extractable organic fluorine using HR-GFMAS. Error bars represent standard deviations (*n* = 3)

A fluorine mass balance analysis was performed to estimate the levels of unidentified organic fluorine compounds (UOF) that resulted from the degradation of GenX or another PFAS or other fluorinated compounds such as pesticides and pharmaceuticals occurring in the soil. The study determined the extractable organic fluorine using HR-GFMAS and percentage contributions of the total PFAS, including the GenX in the rice, wheat, porewater and soil (Figs. 5, 6 and 7).

An LC-MS/MS analysis was performed to identify the PFAS in the rice and wheat shoots, soil and porewater. No other PFAS, apart from GenX, was detected among the 35 targeted PFAS, which included PFCA and PFSA with perfluorocarbon chain lengths of C4-C12, as well as PFAS sulphonamides (Table S1). Notably, ultra-short PFCAs were not monitored in this study due to challenges in their measurement.

GenX at low and high exposure levels was found to be the major contributor to the EOF in the flooded rice shoots, contributing to 78 to 97% of the extractable organic fluorine. In the non-flooded rice shoots, it contributed to 68 to 95% at both exposure levels. The difference in mass balance between flooded and non-flooded conditions was not significant (*P* = 0.133), as well as between different exposure levels (*P* value = 0.103).

In the wheat shoots, GenX was found to contribute to only 50 to 72% of the total extractable fluorine at both low and high exposure levels. Similarly, in the flooded soil, GenX contributed to 64 to 84% of the extractable fluorine, and in non-flooded rice soil, it contributed to 21 to 92% at both exposure levels. While in non-flooded wheat soil contributed to 57 to 97% of the extractable fluorine. The difference in mass balance between flooded and non-flooded conditions was also not significant (*P* value = 0.176), but significant between different exposure levels (*P* value = 0.03). GenX was also found to account for 53 to 94% of the total extractable organic fluorine in non-flooded soil porewater samples, while in flooded soil porewater, it accounted for 53% and 98% at low and high exposure levels, respectively. This difference in mass balance between flooded and non-flooded conditions was not significant (*P* value = 0.447), as well as between different exposure levels (*P* value = 0.480).

## Discussion

### Effects of GenX on the growth and biomass of rice and wheat

The significant reductions of growth parameters shoot length and dry biomass of wheat and rice (Fig. 1) after the 30-day exposure to GenX at the rates of 0.4 and 2.0 mg kg^−1^ are supported by earlier hydroponic study of Chen et al. (2020). They reported that plant species *A. thaliana* and *N. benthamiana* had no growth issues up to 5 mg L^−1^ GenX. However, when GenX dose increased beyond this level, shoot growth was affected negatively. A severe hinderance in the growth and development of the shoots and roots of *N. benthamiana was* observed when exposed to the level of GenX 20 mg L^−1^ GenX. These authors also calculated tolerance index values as 100%, 55% and 40% for control, 0.4 and 2.0 mg kg^−1^, respectively. Another investigation suggested that phytotoxicity as measured through dry mass production of shoot of lettuce of GenX may not appear at concentration of 100 μg L^−1^ (Wang et al. 2023). Hence, it is quite clear that as a first negative effect of GenX, the root development of plants is retarded. Resultantly, the uptake of water and nutrients becomes limited. Consequently, normal growth of above ground parts of the plants is not possible. Another probable effect of GenX within plant tissues could be reduction of photosynthesis. It has been reported that at concentrations between 20 and 200 mg L^−1^, GenX inhibited plant growth through lowering of chlorophyll content and enzyme activity (Chen et al. 2020). Apparently, depending upon the exposed level of GenX, shoot growth, biomass and yield of crops are expected to decrease. The results demonstrate that the rice shoots’ dry biomass significantly reduced under 2.0 mg g^−1^ compared to the dry shoot biomass of wheat (Fig. 1). The growth of the rice was more inhibited in non-flooded soil than was wheat, indicating that rice is more sensitive to GenX than wheat. The variation of different species in their tolerance to biotic and abiotic stresses are well known. These findings suggest that the effects of GenX on plant growth and development are concentration and species-specific.

### Uptake of GenX

When the quantities of GeneX increased in a soil its resultant uptake in plant tissues also increased (Figs. 2 and 4). It means that broadly a proportional uptake of GenX present in different soils was observed in case of rice and wheat. More elaborately, the uptake data revealed that if the level of GenX in a soil is high, its uptake in plant tissues will also be more as compared to a soil having lower quantities of GenX. However, plant specificity is another factor affecting the uptake of GenX because each specie behaves differently to abiotic stresses like GenX. They exercise ion selectivity phenomenon to respond any stress. Thus, uptake and bioaccumulation does not only depend on GenX properties and chemical characteristics but also on plant species and its capacity for bioaccumulation. As Doucette et al. (2018) noted that the differences in PFAS uptake among species are due to varying biotransformation capacities, rates of dissipation, ease of root uptake and temperature. Further, Zhao et al. (2016) found that uptake of PFCA by wheat increased by 1.5- to 2.3-fold when the temperature increased from 20 to 30 °C. In the present study, the rice and wheat were grown in different greenhouses with different temperatures. The rice was grown at 28 °C, while the wheat was grown at 15 °C. Besides other factors, temperature differences may also have impacted the uptake of GenX by wheat and non-flooded rice due to increase in nutrient diffusion rates and photosynthesis activity which is giving more energy. This may also increase the uptake of pollutants (Zhao et al. 2016*)*.

Soils are ultimate carrying and exchange media for all the constituents accumulated in it under natural processes or added by manual activities (Li et al. 2023). The data indicated that soils conditions also affected the uptake and bioaccumulation of GenX in rice and wheat, in addition to the plant species and chemical characteristics of GenX. Particularly clay mineralogy plays an important role in this regard (Schumm et al. 2023) because all the exchange reactions and holding of ions are occurring on the exchange sites of clay minerals present in a soil. When rice was grown in non-flooded soil, higher concentrations of GenX compared to the flooded soil conditions were observed (Fig. 2). The reason may be the dilution and translocation effect water under flooded conditions. Some of the GenX ions might leached down with excessive water to lower soil depths that were not sampled and analysed. This observation agrees with the findings of Lesmeister et al. (2021) who also recorded similar data. They explained such results because of efficient transpiration facilitates and translocation of more molecules through the water gradient. Similarly, flooding of water can affect the distribution of any substance (GenX in the present case) which is water soluble. It is quite possible that GenX could be removed and taken away from the rooting zones of plants. Moreover, lower concentration of GenX dilution effect of excessive water may cause lower intake by roots and further translocation to plant tissues. Resultantly, the uptake of GenX by rice under flooded conditions is lower than non-flooded fields. Suggestions are there as well that the total amount of transpired water during growth of a certain plant can explain the various absorption and translocation abilities of crops (Blaine et al. 2014).

Anaerobic (flooded) soil conditions cause the soil to exhibit different biological and chemical characteristics as compared to analogous non-flooded aerobic soils (Hazra et al. 2023). For example, there is less oxygen in flooded soil because almost all the soil pores are filled with water. Mostly the reduced chemical forms of compounds predominate in the absence of oxygen as well as anaerobic bacterial activities. As a result of such microbial metabolism, the conversion of oxidised elements to their corresponding reduced forms occurs under anaerobic conditions. Enough oxygen is available for bacterial metabolism in aerobic non-flooded soils. Hence, the compounds stay in their oxidised forms. However, the degradation of GenX has not been studied in much depth and needed to investigate systematically. Yet, study of Harfmann et al. (2021) on the sorption of GenX on the sediments can be quoted. It was found that within 14 days, the concentration of GenX in freshwater and estuary sediments decreased by 40 to 59%, yet it was not certain that this decrease was due to biological degradation or not.

### GenX mechanism of intoxication and growth inhibition

Despite the different uptake and bioaccumulation capacities, GenX was found to inhibit the growth of shoot biomass more in rice. GenX, like other PFASs, initiates a mechanism that inhibits shoot growth in plants, but this appears to be species-specific at the concentration used in this study. Our findings are in line with studies involving both human cells in vitro and plant species. Such studies have concluded that exposure to GenX induces an intracellular toxicity mechanism that leads to apoptosis and a reduction of cell viability (Yoo et al. 2021). Similarly, GenX uptake has been observed to have an adverse effect on lettuce even at low concentration levels (100 μg L^−1^), which suggests that GenX could induce oxidative stress in lettuce plants (Wang et al. 2023). The H_2_O_2_ content in lettuce tissues revealed that GenX causes stronger oxidative stress than does PFOA. Previous research also found that 100 to 200 mg L^−1^ of GenX increased H_2_O_2_ in *Nicotiana benthamiana* plants. In our study, we however did not measure the oxidative stress but recorded the growth inhibition by the application of GenX.

### Impact of soil conditions on GenX bioaccumulation

Apart from the plant species and chemical properties of GenX, soil conditions also impacted the uptake and bioaccumulation of GenX in rice. In this experiment, the rice grown in non-flooded soil had higher concentrations of GenX compared to that grown in flooded soil (Fig. 2). This observation is in line with findings from studies that revealed that efficient transpiration facilitates the translocation of more molecules through the water gradient (Lesmeister et al. 2021). Similarly, it has been established that flooding reduces transpiration rate, thereby affecting the distribution of the substance located in the soil and the plant tissues. In non-flooded conditions, the rate of transpiration is generally higher than in flooded conditions, which can lead to higher rates of PFAS uptake by plants. This causes less GenX to be concentrated in the flooded plant comparing to the non-flooded one. It has been suggested that the amount of water that transpires during growth can explain the various absorption and translocation abilities among crops (Blaine et al. 2014).

### TF

Higher TF values were found at higher exposure levels, indicating possibilities of plants to take up more GenX with increasing exposure levels. The TF values for GenX in rice shoots were higher in non-flooded soil than in flooded soil conditions. Zhang et al. (2021) also found that among the five replacement PFAS (ether-PFAS) studied, GenX had the highest TF value in wetland plants (*C. comosa*) exposed to 500 ng L^−1^ for 52 days, which indicates that GenX can be translocated easily to shoots of a wetland plant. However, the dilution effect of excessive water in the flooded soils is very important phenomenon to be considered. The prevailing water in the vicinity of roots must be less concentrated in flooded soil, thus reducing chances of more uptake of GenX. This can be supported by the data of this study because the higher concentration of GenX in the soil porewater under non-flooded conditions were recorded compared to flooded conditions (Fig. 3). This difference in concentration of GenX in the soil porewater can be attributed to the dilution effect of excessive water in flooded soil conditions. Recent studies also reported that short-chain PFAS accumulate more easily in the edible parts of plants compared to long-chain PFAS (Gu et al. 2023; Wang et al. 2023). Overall, in both the flooded and non-flooded rice, TF was statistically different between the GenX treatments. This suggests that the increase in GenX exposure impacted the crop’s efficiency of GenX uptake as well.

### Fluorine mass balance and EOF

The fluorine mass balance analysis results showed that higher exposure levels of GenX significantly contributed to the total extractable fluorine. These results suggest that a close fluorine mass balance exists at high exposure levels, and that most of the extractable fluorine can be accounted for by the presence of GenX. The high contribution of GenX to the total extractable fluorine in the rice shoots, porewater and soil implies no GenX degradation in the plants. However, discrepancies between the EOF and GenX concentrations in the soil at low exposure levels suggest that GenX may degrade to other short-chain PFCAs that were not included in the targeted LC-MS/MS analysis. A recent study examined the effects of fluoroalkylether compounds, including GenX, on the microbial community in soil–plant systems. The results of the study showed that the structure of the community and species diversity were significantly impacted by ether-PFAS at concentrations of 500 ng L^−1^ and 2000 ng L^−1^ (Jiang et al. 2021). Although the study did not find any evidence of biodegradation of the spiked ether-PFAS, it revealed that the presence of these compounds seemed to stimulate the growth of certain microbes with the ability to break down hydrocarbon chemicals and contaminants. In our study, it is significant that at low exposure level, the mass balance between GenX and EOF shows unaccounted organofluorines, which however were not detected in the targeted LC-MS/MS analysis. The unaccounted organofluorines might however be ultra-short-chain PFAS as degradation products which were not monitored. It is important to note that our study did not aim to investigate the mechanisms or pathways of GenX degradation in these plants. Therefore, further research is required to understand the course of this compound in agricultural environments comprehensively in the field.

### GenX distribution coefficient

In this study, the distribution of the substance molecules was determined using the distribution coefficient *K*_*d*_.

An earlier study assessed the behaviours of a wider selection of PFAS on soils of different properties. The study found that per- and polyfluoroalkyl ether acids, including GenX, have low *K*_*d*_ values, which means that they are very mobile. This indicates the hydrophilic nature (hydrophilicity) of these compounds and the ease by which they may be taken up in groundwater to pollute waterways and the environment (Aro et al. 2021).

As indicated in Table 2, the present study showed that *K*_*d*_ values for GenX in rice were influenced by the treatment conditions (flooded vs. non-flooded) and exposure levels. The lower *K*_*d*_ value for GenX suggests a higher degree of mobility, allowing it to be present in the water phase instead of being adsorbed onto the soil particles. In this study, it has been found that GenX had the lowest *K*_*d*_ value in non-flooded conditions, especially at low exposure levels, which suggests that it is more likely to be taken up by rice under non flooding conditions compared to flooded conditions. In other words, the mobility of GenX was higher under non-flooded conditions, resulting in its increased presence in the aqueous phase. This characteristic makes it more available for plant uptake by rice, resulting in a higher likelihood of accumulation. Hence, the results of this study suggest that the risk of GenX accumulation in rice is higher under non-flooded conditions, particularly at low exposure level. These findings are in line with previous evidence indicating that short-chain PFASs like GenX are more readily absorbed through a water gradient and anion channels when in adequate concentrations in the growth medium (Li et al. 2022).

To the best our knowledge, the uptake mechanisms of new alternatives like GenX in plants have not been well studied. Gu et al. (2023) investigated the differences between PFOA and its alternatives, finding that PFOA and GenX were likely transported via a diffusion process. Water channels only have a limited effect on the uptake of PFOA and its alternatives, and slow anion channels rather than rapid ones were mainly responsible for the uptake of PFCAs. If this also applies to rice and wheat, the difference in GenX uptake of the plant species in our experiment can therefore not be a temperature effect but rather a plant species effect.

As observed previously, despite being industrially used as a short-chain alternative to PFOA, GenX still has similar properties that make it unsafe for the environment. According to recent research, PFASs such as GenX accumulate to different degrees in crops, depending on the components of said crops (Krippner et al. 2015; Bizkarguenaga et al. 2016). PFASs have been reported to accumulate in protein-rich plant parts; as a result, GenX may accumulate in the bran and germ of rice and wheat. Although wheat and rice are the model crops for this study, other findings raise concerns about the safety of crops whose shoots are consumed directly, such as lettuces. For instance, in China, water scarcity has shifted from rice cultivation in traditional high-water-consuming lowlands to controlled and non-flooded irrigation approaches. Although non-flooded conditions have been tested successfully for the reduction of other contaminants of concern, such as arsenic (as rice grown in traditional flooded paddies has been found to contain arsenic levels 10 to 15 times greater than rice cultivated in non-flooded conditions (Li et al. 2019)), here we observed higher GenX concentrations in rice shoots grown under non-flooded conditions. This may increase safety risks for crops cultivated under water stressed and rainfed areas.

In contrast to the available published information on PFAS and PFOA substances (Li et al. 2021), this study’s findings are crucial because they demonstrate that despite being a short-chain alternative to PFOA, GenX exhibits chemical properties and environmental safety concerns. Even more than long-chain PFAS (like PFOA), the industrial use of which has been replaced, GenX has the potential to impede plant growth. Studies in line with our findings on shoot biomass and growth length indicate that due to GenX’s high solubility in water, it can enter the environment and affect plants more easily than other PFAS (Ahrens et al. 2010, 2011). Prior studies in rice paddy fields, which are consistent with the present flooded soil conditions, have determined that due to their high mobility in water, short-chain PFAS like GenX are likely to leach into groundwater systems, thereby reducing their availability for plant uptake (Eun et al. 2022). This explains our observation that less GenX accumulated in the shoots of rice grown in flooded soil. Based on these study findings, GenX is not better for the environment because of its higher bioaccumulation and poor degradation. Further, GenX is only poorly absorbed in flooded conditions and better taken up by the rice under non-flooded conditions, making it a concern for modern crop irrigation approaches that aim to optimise production with minimal water use.

## Conclusions

In the present study, we assessed the bioaccumulation potential of GenX, an emerging fluorinated ether or replacement PFAS in wheat and rice at two concentrations. The results demonstrated that GenX was taken up by plant roots, translocated to shoots and accumulated in the tissues of the studied plants. Rice had the highest GenX concentration in its shoots of the two plant species investigated. The accumulation of GenX in rice and wheat increased when the plants were exposed to higher levels of GenX, indicating that rice and wheat growing in hot spots of GenX contamination would be far more affected, as suggested by the low-level exposure experiments. The results of this study indicated that rice is more sensitive to GenX than wheat at relatively high exposure levels. Moreover, the presence of GenX might pose similar or even higher ecotoxicological risks to the environment than PFOA. Long-term PFAS accumulation in surface and wastewater, as well as the potential risk to organisms due to environmental persistence, must be considered seriously and dealt with effectively to reduce adverse environmental consequences. Therefore, it is important to conduct additional research to compare the accumulation and phytotoxic effects of GenX with other plant species and other PFAS under longer exposure times.

### Supplementary information

ESM 1: Tables S1-S6 and Figures S1, S2 (DOCX 48 kb)

## Data Availability

Most data are made available in the supplementary material. Additional data will be made available from the corresponding author on request.
