# Supplementary material for: GenX uptake by wheat and rice in flooded and non-flooded soils: a greenhouse experiment
Source: Environ Sci Pollut Res Int. 2023 Dec 4;31(1):1607–20. doi: 10.1007/s11356-023-31160-w (PMC10789845; doi:10.1007/s11356-023-31160-w)
Supplement: Supplementary file 1 — Tables S1-S6 and Figures S1, S2 (DOCX 48 kb) [file 11356_2023_31160_MOESM1_ESM.docx]

**GenX uptake by wheat and flooded and non-flooded rice: greenhouse experiment.**

Amnah Al Zbedy^1,2,3^, Viktoria Müller^4,5^, Andrew Kindness^5,6^, Rainer Ebel^2^, Gareth J Norton^1^, Joerg Feldmann^4,*^

^1^School of Biosciences, University of Aberdeen, Machar Drive, Aberdeen, AB24 3FX, UK

^2^School of Physical Sciences, University of Aberdeen, Meston Walk, Aberdeen, AB24 3UE, UK

^3^Department of Chemistry, Al-Qunfudhah University College, Umm Al-Qura University, Makkah, Saudi Arabia

^4^TESLA- Analytical Chemistry, University of Graz, Universitätsplatz 1, 8010 Graz, Austria

^5^The James Hutton Institute, Craigiebuckler, Aberdeen, AB15 8QH, UK

^6^School of Chemistry and Physics, University of KwaZulu-Natal, Private Bag X54001, Durban 4000, South Africa

*corresponding author: TESLA- Analytical Chemistry, University of Graz, Universitätsplatz 1, 8010 Graz, Austria, [joerg.feldmann@uni-graz.at](mailto:joerg.feldmann@uni-graz.at)

Supplementary material

Table S1. Physicochemical properties of soil for pot experiment

| Ca meq/100g | Mg meq/100g | Na meq/100g | K meq/100g | pH | N  % | CLAY % | SILT % | SAND % |
| --- | --- | --- | --- | --- | --- | --- | --- | --- |
| 9.09 | 0.62 | 0.11 | 0.28 | 7.30 | 0.42 | 14.45 | 30.14 | 54.21 |

| Table S2. List of analytes and their acronyms |  |  |
| --- | --- | --- |
| Name | Acronym | Carbon number |
| **PFAS carboxylic acids** | **PFCAs** |  |
| Perfluorobutanoic acid | PFBA | C_4_ |
| Perfluoropentanoic acid | PFPA | C_5_ |
| Perfluorohexanoic acid | PFHxA | C_6_ |
| Perfluoroheptanoic acid | PFHpA | C_7_ |
| Perfluorooctanoic acid | PFOA | C_8_ |
| Perfluorononanoic acid | PFNA | C_9_ |
| Perfluorodecanoic acid | PFDA | C_10_ |
| Perfluoroundecanoic acid | PFuDA | C_11_ |
| Perfluorododecanoic acid | PFdDA | C_12_ |
| Perfluorotridecanoic acid | PFtrDA | C_13_ |
| Perfluorotetradecanoic acid | PFteDA | C_14_ |
| Perfluorohexadecanoic acid | PFhxDA | C_16_ |
| Perfluorooctadecanoic acid | PFoDA | C_18_ |
|  |  |  |
| **PFAS sulfonic acids** | **PFSAs** |  |
| Perfluoro-1- butanesulfonic acid | PFBS | C_4_ |
| Perfluoro-1-pentanesulfonic acid | PFPS | C_5_ |
| Perfluoro-1-hexanesulfonic acid | PFHxS | C_6_ |
| Perfluoro-1-heptanesulfonic acid | PFHpS | C_7_ |
| Perfluoro-1-octanesulfonic acid | PFOS | C_8_ |
| Perfluoro-1-nonanesulfonic acid | PFNS | C_9_ |
| Perfluoro-1-decanesulfonic acid | PFDS | C_10_ |
| Perfluoro-1-dodecanesulfonic acid | PFDoS | C_12_ |
|  |  |  |
| **PFAS sulfonamides and sulfonamidoacetic acids** |  |  |
| Perfluoro-1-octanesulfonamide substances | PFOSA |  |
| Perfluorooctanesulfonamidoacetate | PFOSAA |  |
| *N*-ethyl perfluorooctyl sulfonamide | *N*-EtFOSA |  |
| 2-(*N*-Methylperfluoro-1-octanesulfonamido)-ethanol | *N*-MeFOSE |  |
| 2-(*N*-ethylperfluoro-1-octanesulfonamido)-ethanol | *N*-EtFOSE |  |
|  |  |  |
| **Others** |  |  |
| 4,8-Dioxa-3H-perfluorononanoic acid | ADONA |  |
| Hexafluoropropylene oxide dimer acid | GenX |  |
| Perfluorooctylphosphoric acid | PFOPA |  |
| 2-Perfluorooctyl ethanoic acid | FOEA |  |
| Na 8-Chloroperfluoro-1-octansulfonate | 8Cl-PFOS |  |
| Sodium 1H, 1H, 2H, 2H-perfluorodecane sulfonate | 8:2 FTS |  |
| Na 1H, 1H, 2H, 2H-perfluorododecane sulfonate | 10:2 FTS |  |

| Table S3. List of monitored transitions and MS parameters. | | | | |  |  |  |
| --- | --- | --- | --- | --- | --- | --- | --- |
| **Compound Name** | **Precursor Ion** | **Product Ion** | **Ret Time (min)** | **Fragmentor** | **Collision Energy** | **Cell Accelerator Voltage** | **Polarity** |
| 10_2FTS | 627 | 607 | 14.1 | 196 | 52 | 4 | Negative |
| 10_2FTS | 627 | 81 | 14.1 | 196 | 92 | 4 | Negative |
| 8_2FTS | 527 | 507 | 11.4 | 176 | 48 | 7 | Negative |
| 8_2FTS | 527 | 81 | 11.4 | 176 | 80 | 7 | Negative |
| 8Cl-PFOS | 515 | 99 | 13.3 | 202 | 104 | 4 | Negative |
| d7-N-MetFOSE | 623 | 59 | 21.2 | 106 | 48 | 4 | Negative |
| FOEA | 477 | 393 | 10.2 | 96 | 56 | 4 | Negative |
| FOSAA | 556 | 498 | 11.6 | 194 | 44 | 6 | Negative |
| FOSAA | 556 | 419 | 11.6 | 194 | 52 | 6 | Negative |
| GenX | 285 | 169 | 6.1 | 70 | 12 | 5 | Negative |
| GenX | 285 | 119 | 6.1 | 70 | 84 | 8 | Negative |
| M2PFOA | 415 | 370 | 9.2 | 70 | 16 | 4 | Negative |
| M2PFteDA | 715 | 670 | 17.5 | 122 | 36 | 4 | Negative |
| M3PFBA | 216 | 172 | 1.9 | 68 | 12 | 4 | Negative |
| M3PFBS | 302 | 80 | 5.6 | 162 | 88 | 4 | Negative |
| M3PFHxS | 402 | 80 | 9.8 | 176 | 108 | 4 | Negative |
| M4PFHpA | 367 | 322 | 7.5 | 74 | 20 | 4 | Negative |
| M5PFHxA | 318 | 273 | 5.3 | 68 | 28 | 4 | Negative |
| M5PFPA | 268 | 223 | 2.9 | 70 | 12 | 4 | Negative |
| M6PFDA | 519 | 474 | 12.1 | 106 | 16 | 6 | Negative |
| M7PFuDA | 570 | 525 | 13.4 | 82 | 24 | 5 | Negative |
| M8PFOA | 421 | 376 | 9.2 | 68 | 8 | 4 | Negative |
| M8PFOS | 507 | 80 | 12.9 | 196 | 100 | 6 | Negative |
| M9PFNA | 472 | 427 | 10.7 | 84 | 12 | 6 | Negative |
| MPFBA | 217 | 172 | 1.9 | 64 | 8 | 4 | Negative |
| MPFDA | 515 | 470 | 12.1 | 72 | 20 | 6 | Negative |
| MPFdDA | 615 | 570 | 14.8 | 118 | 24 | 6 | Negative |
| MPFOS | 503 | 80 | 12.9 | 204 | 112 | 6 | Negative |
| NaDONA | 377 | 251 | 8.1 | 72 | 20 | 6 | Negative |
| NaDONA | 377 | 85 | 8.1 | 72 | 72 | 6 | Negative |
| N-EtFOSA | 526 | 219 | 20.9 | 168 | 72 | 5 | Negative |
| N-EtFOSA | 526 | 169 | 20.9 | 168 | 108 | 5 | Negative |
| N-EtFOSE | 630 | 59 | 20.8 | 102 | 36 | 5 | Negative |
| N-MetFOSE | 616 | 59 | 20.4 | 106 | 48 | 4 | Negative |
| PFBA | 213 | 19 | 1.9 | 72 | 64 | 4 | Negative |
| PFBA | 213 | 169 | 1.9 | 72 | 12 | 4 | Negative |
| PFBS | 299 | 99 | 5.6 | 158 | 88 | 4 | Negative |
| PFBS | 299 | 80 | 5.6 | 158 | 112 | 4 | Negative |
| PFDA | 513 | 469 | 12.1 | 92 | 20 | 6 | Negative |
| PFDA | 513 | 219 | 12.1 | 92 | 24 | 6 | Negative |
| PFdDA | 613 | 569 | 14.8 | 104 | 24 | 6 | Negative |
| PFdDA | 613 | 269 | 14.8 | 104 | 52 | 6 | Negative |
| PFdDS | 699 | 99 | 18.3 | 208 | 112 | 4 | Negative |
| PFdDS | 699 | 80 | 18.3 | 208 | 116 | 4 | Negative |
| PFDS | 599 | 99 | 15.7 | 214 | 116 | 4 | Negative |
| PFDS | 599 | 80 | 15.7 | 214 | 104 | 4 | Negative |
| PFHpA | 363 | 319 | 7.5 | 74 | 16 | 4 | Negative |
| PFHpA | 363 | 169 | 7.5 | 74 | 28 | 4 | Negative |
| PFHpS | 449 | 99 | 11.4 | 200 | 120 | 4 | Negative |
| PFHpS | 449 | 80 | 11.4 | 200 | 92 | 4 | Negative |
| PFHxA | 313 | 269 | 5.3 | 72 | 12 | 4 | Negative |
| PFHxA | 313 | 119 | 5.3 | 72 | 88 | 4 | Negative |
| PFhxDA | 813 | 769 | 19.1 | 136 | 40 | 4 | Negative |
| PFhxDA | 813 | 169 | 19.1 | 136 | 64 | 4 | Negative |
| PFHxS | 399 | 99 | 9.8 | 186 | 76 | 4 | Negative |
| PFHxS | 399 | 80 | 9.8 | 186 | 108 | 4 | Negative |
| PFNA | 463 | 419 | 10.7 | 84 | 16 | 6 | Negative |
| PFNA | 463 | 219 | 10.7 | 84 | 28 | 6 | Negative |
| PFNS | 549 | 99 | 14.2 | 214 | 120 | 5 | Negative |
| PFNS | 549 | 80 | 14.2 | 214 | 108 | 5 | Negative |
| PFOA | 413 | 369 | 9.1 | 76 | 16 | 4 | Negative |
| PFOA | 413 | 169 | 9.1 | 76 | 28 | 4 | Negative |
| PFoDA | 913 | 869 | 20 | 142 | 52 | 4 | Negative |
| PFoDA | 913 | 169 | 20 | 142 | 96 | 4 | Negative |
| PFOPA | 499 | 79 | 18.2 | 164 | 108 | 5 | Negative |
| PFOS | 499 | 99 | 12.9 | 208 | 104 | 6 | Negative |
| PFOS | 499 | 80 | 12.9 | 208 | 108 | 6 | Negative |
| PFOSA | 498 | 78 | 18.2 | 174 | 92 | 5 | Negative |
| PFPA | 263 | 219 | 2.9 | 56 | 8 | 4 | Negative |
| PFPS | 349 | 99 | 7.9 | 164 | 84 | 4 | Negative |
| PFPS | 349 | 80 | 7.9 | 164 | 108 | 4 | Negative |
| PFteDA | 713 | 669 | 17.5 | 94 | 32 | 4 | Negative |
| PFteDA | 713 | 169 | 17.5 | 94 | 116 | 4 | Negative |
| PFtrDA | 663 | 619 | 16.2 | 104 | 32 | 4 | Negative |
| PFtrDA | 663 | 169 | 16.2 | 104 | 68 | 4 | Negative |
| PFuDA | 563 | 519 | 13.4 | 52 | 16 | 5 | Negative |
| PFuDA | 563 | 169 | 13.4 | 52 | 92 | 5 | Negative |
|  |  |  |  |  |  |  |  |

| Table S4. HPLC method | |  |  |  |  |  |  |
| --- | --- | --- | --- | --- | --- | --- | --- |
| Time (min) | 5 mM ammonium acetate in water (%) | acetonitrile (%) |  |  |  |  |  |
| 0.0 | 75 | 25 |  |  |  |  |  |
| 1.0 | 75 | 25 |  |  |  |  |  |
| 15.0 | 40 | 60 |  |  |  |  |  |
| 19.0 | 0 | 100 |  |  |  |  |  |
| 24.0 | 0 | 100 |  |  |  |  |  |
| 24.1 | 75 | 25 |  |  |  |  |  |
| 28.0 | 75 | 25 |  |  |  |  |  |

Table S5. Temperature program used for the determination of fluorine via molecular absorption of CaF with HR-GFMAS.

| Step | Temperature/ ^o^C | Ramp / ^o^C s^-1^ | Hold/ s |
| --- | --- | --- | --- |
| Dry 1 | 70 | 6 | 15 |
| Dry 2 | 70 | 0 | 5 |
| Pyrolysis | 700 | 300 | 10 |
| Vaporization | 1900 | 3000 | 5 |
| Clean | 2100 | 1000 | 5 |

Table S6. Recovery of the tested PFAS

| PFAS | Recovery (%)  Shoots | Recovery (%)  soil | Recovery (%)  porewater |
| --- | --- | --- | --- |
| MPFBA | 86.0 | 69.0 | 45.6 |
| M5PFPA | 104 | 75.9 | 55.3 |
| M5PFHxA | 82.0 | 56.8 | 52.9 |
| M3PFBS | 107 | 89.3 | 77.2 |
| M4PFHpA | 108 | 73.2 | 70.2 |
| M3PFHxS | 112 | 83.2 | 79.9 |
| M9PFNA | 104 | 69.0 | 69.4 |
| M6PFDA | 103 | 69.4 | 65.7 |
| M8PFOS | 98.8 | 72.9 | 68.0 |
| M7PFuDA | 171 | 106.8 | 89.6 |
| MPFdDA | 71.7 | 65.9 | 41.7 |
| M2PFteDA | 66.2 | 63.4 | 15.3 |

Fig. S1. mass of GenX in rice shoots exposed to GenX at 0.4 mg/kg(low) and 2 mg/kg (high) after 30 days

Fig. S2. mass of GenX in wheat shoots exposed to GenX at 0.4 mg/kg(low) and 2 mg/kg (high) after 30 days.
